# Reducing the probability of radiation-induced hepatic toxicity by changing the treatment modality from helical tomotherapy to fixed-beam intensity-modulated radiotherapy

**DOI:** 10.18632/oncotarget.5581

**Published:** 2015-09-10

**Authors:** Jin Ho Song, Seok Hyun Son, Chul Seung Kay, Hong Seok Jang

**Affiliations:** ^1^ Department of Radiation Oncology, Gyeongsang National University School of Medicine and Gyeongsang National University Hospital, Jinju, Korea; ^2^ Department of Radiation Oncology, Incheon St. Mary's Hospital, College of Medicine, the Catholic University of Korea, Seoul, Korea; ^3^ Department of Radiation Oncology, Seoul St. Mary's Hospital, College of Medicine, the Catholic University of Korea, Seoul, Korea

**Keywords:** radiation-induced hepatic toxicity, hepatocellular carcinoma, helical tomotherapy, fixed-beam intensity-modulated radiotherapy

## Abstract

**Purpose:**

To estimate and compare the risk of radiation-induced hepatic toxicity (RIHT) in helical tomotherapy and fixed-beam intensity-modulated radiotherapy (IMRT) for the treatment of hepatocellular carcinoma (HCC).

**Materials and Methods:**

Twenty patients with unresectable HCC treated with tomotherapy were selected. We performed tomotherapy re-planning to reduce the non-target normal liver volume receiving a dose of more than 15 Gy (NTNL-V_15Gy_), and we created a fixed-beam IMRT plan (FB-P). We compared the dosimetric results as well as the estimated probability of RIHT among the tomotherapy initial plan (T-IP), the tomotherapy re-plan (T-RP), and the FB-P.

**Results:**

Comparing the T-RP and FB-P, the homogeneity index was 0.11 better with the T-RP. However, the mean NTNL-V_15Gy_ was 6.3% lower with the FB-P. These differences result in a decline in the probability of RIHT from 0.216 in the T-RP to 0.115 in the FB-P. In patients whose NTNL-V_15Gy_ was higher than 43.2% with the T-RP, the probability of RIHT markedly reduced from 0.533 to 0.274.

**Conclusions:**

By changing the treatment modality from tomotherapy to fixed-beam IMRT, we could reduce the liver dose and the probability of RIHT without scarifying the target coverage, especially in patients whose liver dose is high.

## INTRODUCTION

Hepatocellular carcinoma (HCC) is one of the most common cancers worldwide, and it is known as the third most common cause of cancer death [1]. Complete resection is still recognized as the most effective treatment in early-stage disease. However, unfortunately, almost 80% of patients present with unresectable disease [2]. Several alternative treatment modalities, such as transarterial chemoembolization, percutaneous ethanol injection, radiofrequency ablation, and radiotherapy have been used in these patients [3].

In the past, the role of radiotherapy in HCC patients was limited because of the poor tolerance of the whole liver to radiation [4]. However, with advances in radiotherapy techniques, such as three-dimensional conformal radiotherapy (3D-CRT), intensity-modulated radiotherapy (IMRT), and helical tomotherapy, several studies have been published reporting the clinical outcomes of these therapies [2, 5–11]. In addition, several clinical and dosimetric parameters have been suggested for predicting the development of radiation-induced hepatic toxicity (RIHT), because the toxicity to the liver is the greatest impediment to improving clinical outcomes [4, 12–19].

IMRT is a powerful technique that can enhance the quality of the dose distribution in some cases by improving the target coverage, the homogeneity of the dose distribution, and the sparing of normal structures [20]. Traditionally, IMRT was delivered by using a linear accelerator with a multi-leaf collimator (MLC) with fixed beams. Compared to the traditional fixed-beam IMRT technique, helical tomotherapy represented by the Hi-ART system (TomoTherapy Inc, Madison, WI) is a type of rotational IMRT in which a 6-megavoltage beam is modulated by binary collimators during continuous rotation [20]. Several reports have been published showing that helical tomotherapy can deliver the same or higher conformal doses to targets while sparing critical organs to a greater extent, such as the head and neck [21, 22]. However, a wide low-dose distribution is one of the disadvantages of this technique, and it can be crucial in the treatment of organs with large volume effects such as the lung and liver [12, 23].

Previously, we reported dosimetric parameters that can predict the probability of RIHT in HCC patients who were treated with helical tomotherapy [18, 24]. In that study, we defined RIHT as an increase of at least 2 points in the Child-Pugh (CP) score within 3 months after the radiation treatment [25], and we concluded that the non-target normal liver receiving a dose of more than 15 Gy (NTNL-V_15Gy_) is the most significant factor for predicting RIHT [18].

In this study, we compared the treatment plans for helical tomotherapy and fixed-beam IMRT in HCC patients. We also defined the extent to which the probability of RIHT could be reduced by changing the treatment modality. Through this study, we could distinguish which patients were good candidates for helical tomotherapy or fixed-beam IMRT.

## RESULTS

### Tomotherapy initial plan (T-IP) vs. tomotherapy re-plan (T-RP)

Several dosimetric parameters are shown in Table 1. The mean NTNL-V_15Gy_ was 47.8% in the T-IP. By performing the re-planning procedure, it was possible to reduce the mean NTNL-V_15Gy_ to 41.1%. This difference was statistically significant (*p* < 0.001). However, the mean dose to the total liver showed no difference between the T-IP and the T-RP (15.3 ± 3.2 Gy vs. 15.6 ± 3.6 Gy, *p* = 0.204).

**Table 1:** Dosimetric comparison between the tomotherapy initial plan (T-IP) and the tomotherapy re-plan (T-RP)

	T-IP	T-RP	*p* value		T-IP vs. T-RP
PTV-D_95%_ (Gy)	41.8 ± 4.0	42.3 ± 3.9	0.070		
CI	1.24 ± 0.11	1.30 ± 0.15	0.008		
HI	1.35 ± 0.11	1.27 ± 0.08	<0.001		
NTNL-V_15Gy_ (mean, %)	47.8 ± 15.1	41.1 ± 14.7	<0.001		
Total liver (mean dose, Gy)	15.3 ± 3.2	15.6 ± 3.6	0.204	QI for liver	1.01
Spinal cord (max dose, Gy)	15.7 ± 7.3	12.2 ± 6.2	<0.001	QI for spinal cord	0.78
Duodenum (mean dose, Gy)	8.4 ± 4.2	7.4 ± 4.4	0.023	QI for duodenum	0.86
Stomach (mean dose, Gy)	9.9 ± 3.9	8.2 ± 3.9	0.011	QI for stomach	0.82
Lt. kidney (mean dose, Gy)	2.1 ± 1.5	2.0 ± 1.4	0.582	QI for Lt. kidney	1.01
Rt. kidney (mean dose, Gy)	4.8 ± 2.8	4.7 ± 3.0	0.793	QI for Rt. kidney	0.99

Abbreviations: PTV-D_95%,_ the dose that 95% of PTV volume received; CI, conformity index; HI, homogeneity index; NTNL-V_15Gy_, the non-target normal liver volume receiving more than 15 Gy, QI, quality index

The target coverage between the T-IP and the T-RP were similar. The PTV-D_95%_ (dose covers 95% of the planning target volume) was slightly higher in the T-RP compared to the T-IP (41.8 ± 4.0 Gy vs. 42.3 ± 3.9 Gy), but without statistical significance (*p* = 0.070). Although the homogeneity index (HI) was slightly better in the T-RP (1.35 ± 0.11 vs. 1.27 ± 0.08, *p* < 0.001), the conformity index (CI) was better in the T-IP (1.24 ± 0.11 vs. 1.30 ± 0.15, *p* = 0.008)

The quality index (QI) was less than 1 for the spinal cord, duodenum, stomach and the right kidney, which means that the organs at risks (OARSs) were better spared in the T-RP. However, the difference was minimal, and the OAR doses satisfied the normal organ dose constraints in both plans.

### Tomotherapy re-plan (T-RP) vs. fixed-beam IMRT plan (FB-P)

Table 2 shows the dosimetric differences between the T-RP and the FB-P. The target dose coverage (PTV-D_95%_) was similar between the T-RP (42.3 ± 3.9 Gy) and the FB-P (42.5 ± 4.1 Gy) (*p* = 0.354). Although the CI slightly improved in the FB-P (1.30 ± 0.15 vs. 1.26 ± 0.07) with no statistical significance (*p* = 0.280), the HI was 0.11 better in the T-RP (1.27 ± 0.08 vs. 1.38 ± 0.19, *p* = 0.006).

**Table 2:** Dosimetric comparison between the tomotherapy re-plan (T-RP) and the fixed-beam IMRT plan (FB-P)

	T-RP	FB-P	*p* value		T-RP vs. FB-P
PTV-D_95%_ (Gy)	42.3 ± 3.9	42.5 ± 4.1	0.354		
CI	1.30 ± 0.15	1.26 ± 0.07	0.280		
HI	1.27 ± 0.08	1.38 ± 0.19	0.006		
NTNL-V_15Gy_ (mean, %)	41.1 ± 14.7	34.8 ± 11.2	<0.001		
Total liver (mean dose, Gy)	15.6 ± 3.6	13.3 ± 3.4	<0.001	QI for liver	0.84
Spinal cord (max dose, Gy)	12.2 ± 6.2	13.6 ± 7.4	0.021	QI for spinal cord	1.12
Duodenum (mean dose, Gy)	7.4 ± 4.4	6.7 ± 4.2	0.077	QI for duodenum	0.91
Stomach (mean dose, Gy)	8.2 ± 3.9	7.0 ± 3.7	<0.001	QI for stomach	0.86
Lt. kidney (mean dose, Gy)	2.0 ± 1.4	1.4 ± 1.0	<0.001	QI for Lt. kidney	0.75
Rt. kidney (mean dose, Gy)	4.7 ± 3.0	3.8 ± 2.6	0.031	QI for Rt. kidney	0.97

Abbreviations: PTV-D_95%_, the dose that 95% of PTV volume received; CI, conformity index; HI, homogeneity index; NTNL-V_15Gy_, the non-target normal liver volume receiving more than 15 Gy, QI, quality index

Despite the loss of homogeneity of the target coverage, the decline of the liver dose was remarkable. The mean NTNL-V_15Gy_ was 34.8% in the FB-P compared to 41.1% in the T-RP. This difference was statistically significant (*p* < 0.001). By changing the treatment modality, we could reduce the NTNL-V_15Gy_ by 6.3%. The mean total liver dose was also 2.3 Gy lower in the FB-P compared to the T-RP (15.6 ± 3.6 vs. 13.3 ± 3.4 Gy) with a QI value of 0.84 (*p* < 0.001).

Other OAR doses were also better in the FB-P, except for the spinal cord. However, the differences in OAR doses between the plans, with the exception of the liver, were small and satisfied the normal organ dose constraints in both plans.

### The probability of RIHT

**Figure 1:**
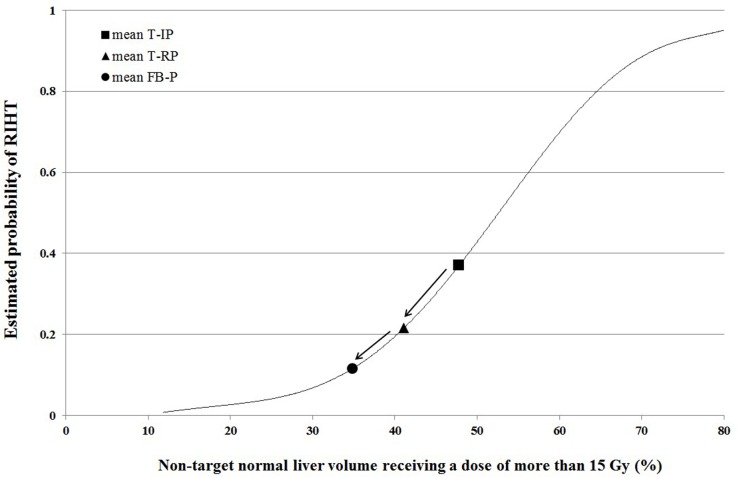
The estimated probability curve of radiation-induced hepatic toxicity (RIHT) for the non-target normal liver receiving a dose of more than 15 Gy (NTNL-V_15Gy_). The mean value of each plan is plotted The tomotherapy initial plan (T-IP, square) has the highest risk of RIHT followed by the tomotherapy re-plan (T-RP, triangle) and the fixed-beam IMRT plan (FB-P, circle).

The mean NTNL-V_15Gy_ of the T-IP, T-RP and FB-P was 47.8%, 41.1%, and 34.8% respectively. These correspond to a probability of RIHT of 0.370, 0.216, and 0.115, respectively. Overall, we could reduce the probability of RIHT by approximately half by changing the treatment modality from helical tomotherapy to fixed-beam IMRT (Figure 1).

In our previous study, we concluded that an NTNL-V_15Gy_ with a cut-off value of 43.2% was the most significant parameter for predicting RIHT [18]. The accuracy was 0.806 with a sensitivity of 0.938 and specificity of 0.725 [18]. We will refer to the patients whose NTNL-V_15Gy_ was higher than 43.2% as the ‘high-risk group’, and all other patients as the ‘low-risk group’. Of 20 patients who were actually treated with T-IP, 10 patients were in the high-risk group and 8 patients actually developed RIHT. In spite of the fact that the NTNL-V_15Gy_ had significantly declined in the T-RP compared to the T-IP, the same 10 patients were still in the high-risk group. However, by changing the treatment plan to FB-P, only 5 patients remained in the high-risk group; 5 patients had moved to the low-risk group.

Another important finding was that the magnitude of the NTNL-V_15Gy_ reduction was different between the risk groups. For the high-risk group patients in the T-RP, we could reduce the NTNL-V_15Gy_ by a mean of 9.9% (53.8% vs. 43.9%) when changing the plan to the FB-P. However, for the low-risk group patients in the T-RP, we could only reduce the NTNL-V_15Gy_ by a mean of 2.6% (28.4% vs. 25.8%). This difference was statistically significant (*p* < 0.001).

The reduction magnitude of NTNL-V_15Gy_ from T-RP to FB-P showed a moderate linear correlation with the NTNL-V_15Gy_ of the T-RP (R^2^ = 0.621, *p* < 0.001). This result indicates that the beneficial effect on the liver dose achieved by changing the plan to fixed-beam IMRT from tomotherapy is greater in patients whose liver dose is higher in the tomotherapy plan.

**Figure 2:**
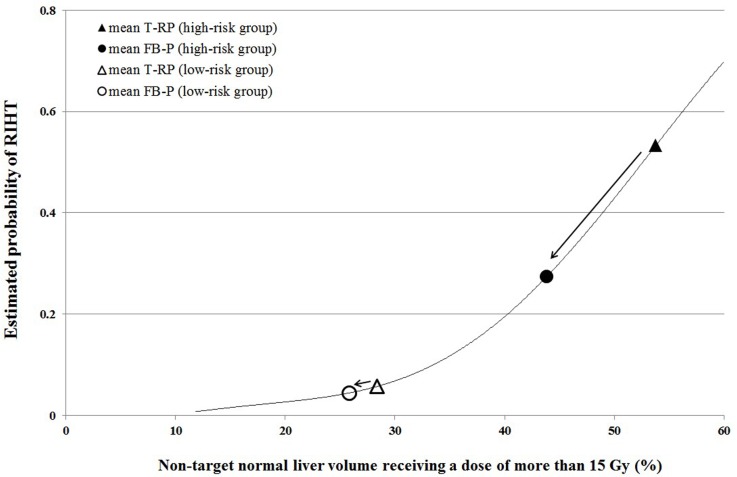
The risk reduction from tomotherapy re-plan (T-RP, triangle) to fixed-beam IMRT plan (FB-P, circle) between risk groups divided by the NTNL-V_15Gy_ of T-RP The risk reduction was much more remarkable in the high-risk group whose NTNL-V_15Gy_ was higher than 43.2% in the tomotherapy plan (filled) compared to low-risk group (empty).

The estimated probability curve of RIHT between the risk groups is shown in Figure 2. If the high-risk group patients were treated with tomotherapy, the probability of RIHT was 0.533. This can be reduced to 0.274 by changing the plan to FB-P. In this group of patients, the probability of RIHT reduced by half. However, the probability of RIHT did not differ much in low-risk group patients. The probability of RIHT reduced from 0.057 to 0.044 by changing the treatment plan from T-RP to FB-P.

## DISCUSSION

In the treatment of HCC patients, radiotherapy had not been used widely because of the low tolerability of the entire liver [4]. Traditionally, it has been shown that 5-10% of patients who receive 30-35 Gy of radiation to the whole liver experience RIHT [12]. However, several recent studies showed that partial irradiation of the liver with higher doses is possible with the advancement of radiation techniques [7–11]. An effective radiation dose can be delivered within an acceptable range of RIHT. However, as RIHT is still the most important dose-limiting factor, several clinical and dosimetric parameters have been reported to predict RIHT [12–19].

Cheng *et al*. analyzed 89 HCC patients who were treated with 3D-CRT. They found that the mean dose to the liver was significantly higher in patients who developed RIHT (22.9 vs. 19.0 Gy, *p* = 0.05) [19]. They also reported that hepatitis B virus status and CP class B were clinical risk factors. Dawson *et al*. also analyzed 203 patients with primary and metastatic liver tumors treated with 3D-CRT and found that a mean dose with a cut-off value of 31 Gy was the most significant factor contributing to RIHT [12]. They also showed that metastatic tumors were more vulnerable than primary liver cancer. Liang *et al*. also reported that the liver volume receiving ≥ 20 Gy (V_20Gy_) was the most significant dosimetric parameter, with a cut-off value of 48.5% [16]. In our previous study based on data from 72 HCC patients who were treated with helical tomotherapy, we concluded that the normal liver volume receiving ≥ 15 Gy (V_15Gy_) was the most significant factor [18]. Not only the various parameters that have been suggested to date, but also the various definitions of RIHT, treatment modalities, and dose schedules make it difficult to find a definite parameter for predicting RIHT. However, the parameters that have been suggested show that while treating the liver with radiation, the large volume effect of the liver is still important [12, 13, 17]. Therefore, reducing the low dose region of the normal liver is crucial in preventing RIHT.

In our study, we showed that by changing the treatment modality from helical tomotherapy to fixed-beam IMRT, one could not only reduce the mean dose to the total liver, but also significantly reduce the NTNL-V_15Gy_. Although we performed a tomotherapy re-planning to reduce the NTNL-V_15Gy_, the mean dose to the liver and the NTNL-V_15Gy_ were still 2.3 Gy and 6.3% lower in the FB-P, respectively, which was statistically significant. By lowering the liver dose, the probability of RIHT also reduced from 0.216 in the T-RP to 0.115 in the FB-P. The magnitude of the liver dose reduction was more remarkable in patients whose liver dose was high in the tomotherapy plan. In high-risk group patients whose NTNL-V_15Gy_ was higher than 43.2% in the tomotherapy plan, the probability of RIHT dropped from 0.533 in the T-RP to 0.274 in the FB-P.

Compared to our study, Hsieh *et al.* reported that helical tomotherapy and non-coplanar IMRT are potentially better than coplanar IMRT for HCC patients with portal vein thrombosis [26]. They documented that tomotherapy showed better uniformity than both IMRT techniques, and they contended the inferiority of coplanar IMRT based on the highest dose of V_30Gy_ in the liver (21% in coplanar IMRT, 17% in tomotherapy and non-coplanar IMRT). However, the V_10Gy_ was also the highest with helical tomotherapy in their study, with statistical significance (72.5% in tomotherapy, 64.8% in coplanar IMRT). Lee *et al.* also suggested that helical tomotherapy showed better CI, HI, and liver doses compared to fixed-beam IMRT in HCC or metastatic liver tumor patients [27]. However, the benefit was limited only in patients with multiple liver tumors. For single tumors, no statistical difference was observed between tomotherapy and fixed-beam IMRT in terms of target coverage and liver doses. A shorter delivery time and lower MU were achieved in the IMRT group. In our study, we evaluated not only the liver dose but also the probability of RIHT. To our knowledge, our study is the only study which compares the radiation techniques on the aspect of complication probability in HCC patients. Our results demonstrated that the reduction of the probability of RIHT was more remarkable than the reduction of the liver dose. As shown in the high-risk group patients, 9.9% reduction of NTNL-V_15Gy_ could reduce the risk of RIHT by half (0.533 vs. 0.274).

In conclusion, it seems clear that by changing the treatment modality from helical tomotherapy to fixed-beam IMRT, we could reduce the liver dose and the probability of RIHT without sacrificing the target coverage or the normal structure doses. In particular, in patients whose liver dose, such as NTNL-V_15Gy,_ is high in a tomotherapy plan, changing the treatment modality to fixed-beam IMRT should be strongly considered to reduce the risk of RIHT.

## MATERIALS AND METHODS

### Patient selection

Between March 2006 and February 2012, 72 patients with unresectable locally advanced HCC were treated with helical tomotherapy at Seoul St. Mary's hospital and Incheon St. Mary's hospital, the Catholic University of Korea. The clinical and dosimetric details were previously reported [18].

**Figure 3:**
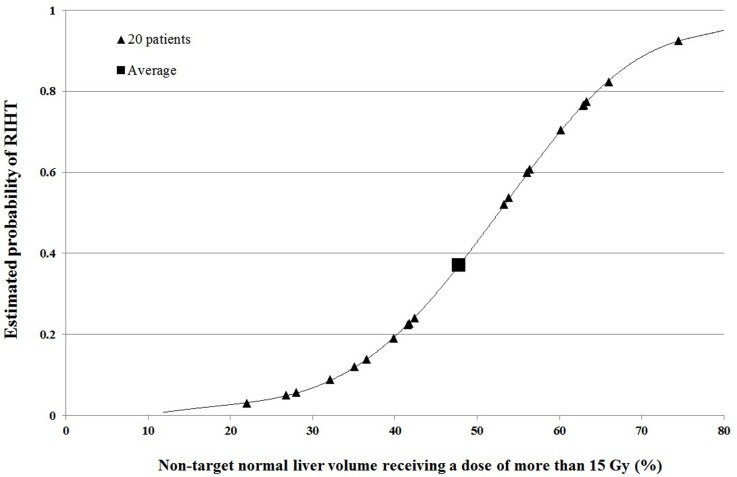
The estimated probability curve of radiation-induced hepatic toxicity (RIHT) for the non-target normal liver receiving a dose of more than 15 Gy (NTNL-V_15Gy_). The data for each patient are plotted with a triangle symbol The mean NTNL-V_15Gy_ was 47.8%, and so the estimated probability of RIHT was 0.370 with the tomotherapy initial plan (T-IP, square).

Of these 72 patients, we selected 20 patients with various probabilities of RIHT as shown in Table 3. The probability of RIHT was calculated based on the NTNL-V_15Gy_, which was the most significant dosimetric parameter for predicting RIHT in our previous study [18]. The estimated probability curve of RIHT and the individual data of each patient are shown in Figure 3. We divided the probability of RIHT into 5 groups with 20% intervals. Although it was impossible to select every four patients evenly, we selected at least 2 patients in every groups. The mean NTNL-V_15Gy_ was 47.8%, so the mean probability of RIHT of these patients was 0.370. The clinical and dosimetric characteristics of these patients are shown in Table 3.

**Table 3:** Patient characteristics

Characteristic	No. of patients	(%)
Gender		
Male	14	(70)
Female	6	(30)
Age (year)	median 66.5	range 48-80
Hepatitis		
No	2	(10)
Yes	18	(90)
HBV	14	(70)
HCV	3	(15)
Others	1	(5)
Child-Pugh class		
A	17	(85)
B	3	(15)
AJCC stage		
II	4	(20)
III	16	(80)
Volume (cm^3^)		
PTV	median, 223.5	range,42-321
NTNL	median, 1002.5	range,704-3636
Prescription dose		
40 Gy / 10 fractions	1	(5)
45 Gy / 10 fractions	2	(10)
50 Gy / 10 fractions	17	(85)
Estimated probability of RIHT		
0 to 20.0%	7	(35)
20.1 to 40.0%	3	(15)
40.1 to 60.0%	3	(15)
60.1 to 80.0%	5	(25)
80.1 to 100.0%	2	(10)

Abbreviations: HBV, hepatitis B virus; HCV, hepatitis C virus; AJCC, American Joint Committee on Cancer; PTV, planning target volume; NTNL, non-target normal liver; RIHT, radiation-induced hepatic toxicity

### Treatment plans

*1. Tomotherapy initial plan (T-IP):* All patients underwent a 3-phase dynamic computed-tomography (CT) scan for simulation. The gross tumor volume (GTV) was contoured as the mass enhanced in the arterial phase and diluted in the delayed phase. No clinical target volume (CTV) was defined, and the planning target volume (PTV) margin was defined individually according to the internal margin acquired from the 4-dimensional CT (4D-CT) data. For some patients who did not undergo 4D-CT, a 5-15 mm margin was added asymmetrically from the GTV. Several OARs were contoured: the total liver, NTNL, stomach, duodenum, both kidneys, and the spinal cord. The NTNL was defined as the volume of the total liver excluding the PTV. A median dose of 50 Gy (range, 40-50 Gy) was prescribed for 95% of the PTV, and it was delivered in 10 fractions. Treatment planning was performed with the built-in software of the TomoTherapy Hi-Art Planning System (TomoTherapy Inc.). All patients were actually treated with this tomotherapy initial plan (T-IP).

*2. Tomotherapy re-plan (T-RP):* We performed a re-planning procedure for helical tomotherapy, as we recently determined that NTNL-V_15Gy_ is the most significant dosimetric parameter for predicting RIHT [18]. The tomotherapy re-plan (T-RP) was designed by using the same TomoTherapy Planning System with the same CT images, same target, and same OARs of T-IP. However, we focused on reducing the NTNL-V_15Gy_ as much as possible, while keeping the target dose coverage at the same level as that of T-IP. The dose constraints for each OAR were determined using the same criteria as that of T-IP.

*3. Fixed-beam IMRT plan (FB-P):* All targets and OARs delineated on the TomoTherapy Planning Station were transferred to the iPlan RT planning system version 4.1.2 (BrainLAB, Feldkirchen, Germany) via the Digital Imaging and Communications in Medicine protocol. The fixed-beam IMRT plan (FB-P) was created for Novalis (BrainLAB) treatment with the same CT images, same target, and same OARs. The plans were designed with an arrangement of a median of 8 beams (range, 6-10). The same dose was prescribed to the 95% isodose volume of the PTV. While achieving the same target coverage, the plan was also designed to reduce the NTNL-V_15Gy_ as much as possible.

### Parameters for plan evaluation & statistical analysis

To compare the dosimetric results between the plans, the following dose-volume histograms (DVH) data and parameters were used.

1. NTNL-V_15Gy_ (%): The NTNL volume that receiving a dose of more than 15 Gy (V_15Gy_), which is the most significant dosimetric parameter for predicting RIHT in our previous study [18].

2. The probability of RIHT: RIHT was defined as an increase of at least 2 points of the CP score within 3 months after the radiation treatment. The probability curve was calculated by using a logistic regression method described in our previous study and is shown in Figure 3 [18].

3. PTV-D_95%_ (Gy): The dose covers 95% of the PTV volume was used to evaluate the target coverage

4. Conformity index (CI): A ratio used to evaluate the tightness of fit of the PTV to the prescription isodose volume [28]. A lower CI value indicates better conformity.

$$\text{CI}=\frac{{\text{V}}_{\text{PTV}}}{{\text{TV}}_{\text{PV}}}\times \frac{{\text{V}}_{\text{TV}}}{{\text{TV}}_{\text{PV}}}$$

[V_PTV_, PTV volume; V_TV_, treatment volume of the prescribed isodose lines; TV_PV_, volume of V_PTV_ within the V_TV_]

5. Homogeneity index (HI): A ratio used to evaluate the homogeneity of the PTV [29]. A lower HI value indicates better homogeneity.

$$\text{HI}=\frac{{\text{D}}_{1\%}}{{\text{D}}_{99\%}}$$

[D_1%_ and D_99%_ are the minimum doses delivered to 1% and 99% of the PTV, respectively]

6. Quality index (QI): An index used to evaluate the difference in the absorbed dose at the OARs. It uses the maximum dose for serial OARs (spinal cord in our study) and the mean dose for parallel OARs [29].

$$\begin{array}{cc} {\text{QI}}_{\text{Serial}}=\frac{{\text{D}}_{\max}^{\text{plan}1}}{{\text{D}}_{\max}^{\text{plan}2}}, & {\text{QI}}_{\text{Parallel}}=\frac{{\text{D}}_{\text{mean}}^{\text{plan}1}}{{\text{D}}_{\text{mean}}^{\text{plan}2}} \end{array}$$

To compare the dosimetric results between the plans, a paired t-test was used. Pearson's correlation analysis was performed to define the factors that influence the probability of RIHT. MedCalc version 14.12 (MedCalc Software bvba, Ostend, Belgium) was used for the statistical analysis, and the differences were considered statistically significant at a *p* value of < 0.05.
